# Association of ocular diseases with schizophrenia, bipolar disorder, and major depressive disorder: a retrospective case-control, population-based study

**DOI:** 10.1186/s12888-020-02881-w

**Published:** 2020-10-02

**Authors:** Chun-Hao Liu, Eugene Yu-Chuan Kang, Yu-Hsiang Lin, Wei-Chi Wu, Zhuo-Hao Liu, Chang-Fu Kuo, Chi-Chun Lai, Yih-Shiou Hwang

**Affiliations:** 1 Department of Psychiatry, Chang Gung Memorial Hospital, Linkou Medical Center, Taoyuan, Taiwan; 2 Department of Psychiatry, New Taipei Municipal Tu-Cheng Hospital, New Taipei, Taiwan; 3 College of Medicine, Chang Gung University, Taoyuan, Taiwan; 4 Department of Sinophone Literatures, National Dong Hwa University, Hualien, Taiwan; 5 Department of Ophthalmology, Chang Gung Memorial Hospital, Linkou Medical Center, Taoyuan, Taiwan; 6 Department of Urology, Chang Gung Memorial Hospital, Linkou Medical Center, Taoyuan, Taiwan; 7 Graduate Institute of Clinical Medical Sciences, College of Medicine, Chang Gung University, Taoyuan, Taiwan; 8 Department of Neurosurgery, Chang Gung Memorial Hospital, Linkou Medical Center, Taoyuan, Taiwan; 9 Department of Rheumatology, Chang Gung Memorial Hospital, Linkou Medical Center, Taoyuan, Taiwan

**Keywords:** Glaucoma, Ocular neurovascular diseases, Psychiatric disorders, Mental disorders

## Abstract

**Background:**

Psychiatric disorders and ocular neurovascular diseases may share a similar pathophysiological route of vascular structures or neurological changes. The aim of this study is to investigate the association between ocular neurovascular diseases and the risk of major psychiatric disorders.

**Methods:**

This was a retrospective case–control, population-based study including patients aged ≥20 and were diagnosed between 1997 and 2013. Ocular neurovascular diseases diagnosed between 1997 and 2006 and newly diagnosed psychiatric disorders including bipolar disorder (BD), major depressive disorder (MDD), and schizophrenia between 2007 and 2013 were registered. Patients were propensity-score matched with control groups without psychiatric disorders in each cohort based on selected covariates.

**Results:**

A total of one million sampled patients in the database were categorized based on their diagnoses; 2243 (37.4% men) were categorized into the BD group, 10,110 (35.2% men) into the MDD group, and 1623 (43.1% men) into the schizophrenia group. In the BD group, all glaucoma (OR 1.49, [1.18–1.89]), open-angle glaucoma (OR 2.08, [1.34–3.24]), and closed-angle glaucoma (OR 2.12, [1.36–3.33]) showed statistical significance of risk. In the MDD group, age-related macular degeneration (OR 1.33, [1.13–1.57]), all glaucoma (OR 1.24, [1.11–1.37]), open-angle glaucoma (OR 1.47, [1.21–1.80]), and dry eye syndrome (OR 1.22, [1.13–1.31]) were associated with a significantly higher risk. In the schizophrenia group, only all glaucoma (OR 1.47, [1.02–2.11]), glaucoma suspect (OR 1.88, [1.01–3.49]), and open-angle glaucoma (OR 2.19, [1.13–4.26]) showed statistical significance.

**Conclusions:**

In this population-based study, ocular neurovascular diseases, especially glaucoma, were associated with increased risks of BD, MDD, and schizophrenia.

## Background

Psychiatric disorders, including schizophrenia, bipolar disorder (BD), and major depressive disorder (MDD), can cause significant global disease burden, disability, and even premature mortality [1, 2]. Although many studies have focused on the pathophysiology of psychiatric disorders, the association between psychiatric disorders and systemic physical conditions is still under investigation. In 2015, a study reported that patients diagnosed with BD or MDD had a tier II moderate risk of cardiovascular disorders in later life [3]. Additionally, BD and MDD were associated with vascular diseases through pathophysiological factors (such as inflammation or endothelial dysfunction), behavioral and environmental factors, and medication-related factors [3, 4].

Microvasculature of the retina is easily observed and shares the same morphological, physiological, and pathological properties as the cerebral vasculature, making the eyes ideal “windows” for evaluating central nervous system disorders [5]. According to previous investigations, retinal vascular change or degeneration was associated with cerebral diseases such as Alzheimer’s and Parkinson’s disease [6, 7]. These associations indicate that we can monitor or screen cerebral diseases through certain ocular conditions. Understanding any association between psychiatric disorders and ocular diseases may lead to the discovery that they also share similar pathophysiological routes of vascular structure or neurological changes.

Previous studies have found some significant retinal changes in patients with psychiatric disorders, especially BD and schizophrenia; both had a higher tortuosity index of retinal arterioles [8] and increased complexity of vascular branching [9]. Another study found that a lower arteriovenular ratio was associated with higher diastolic blood pressure, and a higher arterio-venular ratio was associated with better endothelial function in patients with BD but not in healthy controls [10]. In addition to the microvasculature, the retinal ganglion cell layer also showed some differences in patients with BD and schizophrenia. The retinal ganglion cell layers were thinner in patients with BD, [11] whereas the retinal nerve fiber and ganglion cell layer were both thinner in patients with BD and schizophrenia compared with healthy controls [12, 13]. There has only been a limited study on retinal structure abnormalities in patients with MDD.

The association between psychiatric disorders and ocular diseases is still under investigation; only a few studies have focused on this issue, and the majority of them were cross sectional studies that lacked a large sample size. Because these studies were unable to clarify the association between psychiatric disorders and ocular diseases, we conducted a population-based study to investigate the association between psychiatric disorders and ocular diseases by using a longitudinal design.

## Methods

### Study population

This retrospective case–control study examined the association between ophthalmology diseases and psychiatric disorders. This study was conducted based on the Longitudinal Health Insurance Database 2010 (LHID 2010), which is a subset of the database from the Taiwan National Health Insurance Research Database (NHIRD). The LHID 2010 includes data relating to the insurance claims of one million randomly sampled people from 1997 to 2013. The single-payer Taiwan National Health Insurance (NHI) covered most of the medical expenditure, including inpatient and outpatient services in Taiwan. Because of NHI’s mandatory enrollment and affordability in Taiwan, long-term follow-up is nearly complete. Further information regarding the NHI program and the NHIRD has been reported in previous publications [14–16]. To ensure patient privacy, all identifiable data were encrypted before release; thus, researchers cannot identify individuals from the data. The study was approved by the Chang Gung Memorial Hospital Institutional Review Board (201900967B0).

### Study design

**Fig. 1:**
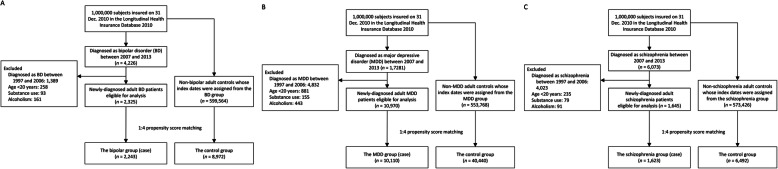
**a** Flow chart of subject selection of bipolar disorder (BD) from the NHIRD. **b** Flow chart of subject selection of major depressive disorder (MDD) from the NHIRD. **c** Flow chart of subject selection of schizophrenia from the NHIRD

In this study, we investigated whether exposure to ocular neurovascular disease in a psychiatric disorder–free cohort increases the risk of psychiatric disorder. We established three study cohorts based on three psychiatric disorders: (1) BD, (2) MDD, and (3) schizophrenia. Patients were respectively identified based on their diagnosis of BD, MDD, or schizophrenia, made between January 1, 2007, and December 31, 2013. Cases were ascertained through three or more diagnoses by a psychiatrist during outpatient visits. The date of the first diagnosis of BD, MDD, or schizophrenia was the index date for the case group. The control group included patients without any diagnosis of BD, MDD, or schizophrenia during the period of our database (1997 to 2013), respectively, in each cohort. The index date of the control group was assigned from that of their counterpart case group. Patients with BD, MDD, or schizophrenia diagnosed between 1997 and 2006; aged less than 20 years; or with a history of substance use or alcoholism were excluded (Fig. 1). Finally, patients who had received a new diagnosis of psychiatric disorders were propensity-score matched with control patients based on selected covariates.

### Exposure to ocular disease and covariates

Within the three cohorts, we recorded ocular neurovascular diseases, including age-related macular degeneration, central serous retinopathy, retinal vascular occlusion, diabetic retinopathy, glaucoma, dry eye syndrome, and optic neuritis, diagnosed between January 1, 1997, and December 31, 2006. To detect the possibility of unmeasured confounding factors, we also identified several negative control exposures to ocular diseases, including retinal detachment, uveitis, and blepharitis [17]. Exposure was ascertained through three or more diagnoses by an ophthalmologist during outpatient visits, and all exposures to ocular disease were censored and counted for the analysis. The covariates included age at the index date, sex, urbanization level, monthly income, comorbidities (anxiety disorder, hypertension, dyslipidemia, diabetes, coronary heart disease, chronic obstructive pulmonary disease, chronic kidney disease, and stroke), and the Charlson Comorbidity Index (CCI) score. Comorbidity was considered based on a minimum of three outpatient diagnoses or one inpatient diagnosis performed between 1997 and the index date. Comorbidities were identified using the International Classification of Diseases, Ninth Revision, Clinical Modification (ICD-9-CM) codes, including outcomes, exposure to ocular disease, and comorbidities (Supplementary Table 1).

### Statistical analysis

To reduce possible confounding factors caused by selection bias, a propensity score matching (PSM) method was used in this study. The propensity score was the predicted probability to be in the case group given the values of covariates and using multivariable logistic regression without considering interaction effects. The variables selected to calculate the propensity score were baseline characteristics (Table 1). Each patient in the case group was matched with four control patients. The matching was processed using a greedy nearest neighbor algorithm with a caliper of 0.2 times the standard deviation of the logit of the propensity score, with random matching order and without replacement. Three separate PSMs were conducted for each of the three psychiatric disorders. The quality of matching was checked using the absolute value of the standardized difference (STD) between the groups, where a value less than 0.1 was considered a negligible difference. The association of each ocular disease and psychiatric disorder was investigated using the generalized estimating equation (GEE), in which the within-pair clustering of outcomes after PSM was accounted for by using a robust standard error and exchangeable working correlation. The link function was logit, and the distribution was binomial in the GEE model. A two-sided *P* value of <.05 was considered statistically significant, and no adjustment for multiple testing (multiplicity) was made in this study. All statistical analyses were performed using SAS version 9.4 (SAS Institute, Cary, NC), including the procedures of “psmatch” for PSM and “genmod” for GEE.

**Table 1:** Characteristics of cases after propensity score matching

	Bipolar Disorder			Major Depressive Disorder			Schizophrenia		
Variable	BD (*n* = 2243)	Control (*n* = 8972)	STD	MDD (*n* = 10,110)	Control (*n* = 40,440)	STD	Schizophrenia (*n* = 1623)	Control (*n* = 6492)	STD
Male	838 (37.4)	3311 (36.9)	0.01	3561 (35.2)	14,126 (34.9)	0.01	699 (43.1)	2753 (42.4)	0.01
Age (years)	43.5 ± 16.1	43.3 ± 15.9	0.01	47.7 ± 16.9	47.3 ± 17.3	0.03	41.9 ± 14.3	42.3 ± 14.5	− 0.03
Urbanization level									
Low	152 (6.8)	583 (6.5)	0.01	781 (7.7)	3137 (7.8)	< 0.01	174 (10.7)	665 (10.2)	0.02
Moderate	584 (26.0)	2384 (26.6)	−0.01	2787 (27.6)	11,135 (27.5)	< 0.01	474 (29.2)	1926 (29.7)	−0.01
High	768 (34.2)	3034 (33.8)	0.01	3373 (33.4)	13,564 (33.5)	< 0.01	494 (30.4)	1969 (30.3)	< 0.01
Very High	739 (32.9)	2971 (33.1)	< 0.01	3169 (31.3)	12,604 (31.2)	< 0.01	481 (29.6)	1932 (29.8)	< 0.01
Monthly income, NTD									
0–17,880	886 (39.5)	3553 (39.6)	< 0.01	3419 (33.8)	13,798 (34.1)	−0.01	920 (56.7)	3673 (56.6)	< 0.01
17,881–22,800	757 (33.7)	3051 (34.0)	−0.01	3373 (33.4)	13,499 (33.4)	< 0.01	485 (29.9)	2035 (31.3)	−0.03
> 22,800	600 (26.7)	2368 (26.4)	0.01	3318 (32.8)	13,143 (32.5)	0.01	218 (13.4)	784 (12.1)	0.04
Comorbidity									
Anxiety disorder	1399 (62.4)	5553 (61.9)	0.01	5307 (52.5)	21,312 (52.7)	< 0.01	655 (40.4)	2626 (40.4)	< 0.01
Hypertension	521 (23.2)	1976 (22.0)	0.03	2918 (28.9)	11,390 (28.2)	0.02	263 (16.2)	1057 (16.3)	< 0.01
Dyslipidemia	409 (18.2)	1532 (17.1)	0.03	2252 (22.3)	8766 (21.7)	0.01	179 (11.0)	715 (11.0)	< 0.01
Diabetes	268 (11.9)	1040 (11.6)	0.01	1351 (13.4)	5213 (12.9)	0.01	136 (8.4)	552 (8.5)	< 0.01
Coronary heart disease	262 (11.7)	973 (10.8)	0.03	1649 (16.3)	6283 (15.5)	0.02	112 (6.9)	481 (7.4)	−0.02
COPD	203 (9.1)	781 (8.7)	0.01	1115 (11.0)	4391 (10.9)	0.01	113 (7.0)	459 (7.1)	< 0.01
Chronic kidney disease	151 (6.7)	593 (6.6)	< 0.01	815 (8.1)	3145 (7.8)	0.01	80 (4.9)	345 (5.3)	−0.02
Stroke	71 (3.2)	267 (3.0)	0.01	441 (4.4)	1643 (4.1)	0.01	47 (2.9)	187 (2.9)	< 0.01
CCI score	0.56 ± 1.13	0.55 ± 1.21	0.01	0.68 ± 1.24	0.62 ± 1.23	0.05	0.39 ± 0.96	0.38 ± 1.01	0.01

*BD* bipolar disorder; *MDD* major depressive disorder; *STD* standardized difference; *OPD* outpatient clinic; *OPH* ophthalmology; *NTD* new Taiwan dollar; *COPD* chronic obstructive pulmonary disease; *CCI* Charlson Comorbidity Index; Data are presented as the frequency (percentage) or the mean ± standard deviation

## Results

### Study cohorts and characteristics

Among one million patients in the LHID 2010, between 2007 and 2013, a total of 4226 patients were diagnosed with BD, 17281 were diagnosed with MDD, and 6073 were diagnosed with schizophrenia. After applying excluding criteria, there were 2325 newly diagnosed BD cases; 10,970 newly diagnosed MDD cases, and 1645 newly diagnosed patients with schizophrenia eligible for analysis. After PSM, 2243 cases and 8972 controls, 10,110 cases and 40,440 controls, and 1623 cases and 6492 controls remained for the BD, MDD, and schizophrenia cohorts, respectively **(**Fig. 1**)**. After PSM, the mean age of cases was 43.5 ± 16.1 years for the BD cohort, 47.7 ± 16.9 years for the MDD cohort, and 41.9 ± 14.3 years for the schizophrenia cohort. All the characteristics were well-balanced between the case and control groups in the 3 study cohorts (Table 1).

### Bipolar disorder cohort

The result of the GEE model showed that previous glaucoma was associated with a higher risk of BD (OR 1.49, 95% CI 1.18–1.89). Among the different types of glaucoma, glaucoma suspect (OR 1.63, 95% CI 1.09–2.43), open-angle glaucoma (OR 2.08, 95% CI 1.34–3.24), closed-angle glaucoma (OR 2.12, 95% CI 1.36–3.33), and undetermined glaucoma (OR 1.46, 95% CI 1.04–2.04) showed significant associations with BD. In addition, there were no associations between the negative control ocular diseases and risk of BD (Table 2).

**Table 2:** Association between each ocular disease and the risk of bipolar disorder (BD)

	Number (%)		Risk of BD	
Ocular Disease	BD (n = 2243)	Control (n = 8972)	OR (95% CI)	*P*
Age-related macular degeneration	29 (1.29)	95 (1.06)	1.22 (0.80–1.86)	0.347
Central serous retinopathy	3 (0.13)	23 (0.26)	0.52 (0.16–1.74)	0.289
Retinal vascular occlusion	4 (0.18)	23 (0.26)	0.70 (0.24–2.01)	0.503
Diabetic retinopathy	37 (1.65)	168 (1.87)	0.88 (0.61–1.26)	0.481
Glaucoma	93 (4.15)	253 (2.82)	1.49 (1.18–1.89)	**0.001**
Glaucoma suspect	34 (1.52)	84 (0.94)	1.63 (1.09–2.43)	**0.017**
Open-angle glaucoma	30 (1.34)	58 (0.65)	2.08 (1.34–3.24)	**0.001**
Closed-angle glaucoma	29 (1.29)	55 (0.61)	2.12 (1.36–3.33)	**0.001**
Undetermined glaucoma	46 (2.1)	127 (1.4)	1.46 (1.04–2.04)	**0.027**
Dry eye syndrome	184 (8.20)	687 (7.66)	1.08 (0.91–1.27)	0.380
Optic neuropathy	1 (0.04)	11 (0.12)	0.36 (0.05–2.82)	0.333
Negative control exposure				
Retinal detachment	5 (0.22)	28 (0.31)	0.71 (0.27–1.85)	0.488
Uveitis	15 (0.67)	67 (0.75)	0.89 (0.51–1.56)	0.696
Blepharitis	70 (3.12)	303 (3.38)	0.92 (0.71–1.20)	0.538

*OR* odds ratio; *CI* confidence interval; *NA* not applicable

### Major depressive disorder cohort

Among the ophthalmologic diseases, the GEE model showed that age-related macular degeneration (OR 1.33, 95% CI 1.13–1.57), glaucoma (OR 1.24, 95% CI 1.11–1.37), and dry eye syndrome (OR 1.22, 95% CI 1.13–1.31) were significantly associated with a higher risk of MDD. Among the different types of glaucoma, glaucoma suspect (OR 1.47, 95% CI 1.22–1.76), open-angle glaucoma (OR 1.47, 95% CI 1.21–1.80), and undetermined glaucoma (OR 1.21, 95% CI 1.05–1.40) were significantly associated with MDD. With respect to the negative control diseases, blepharitis was associated with a lower risk of MDD (OR 0.87, 95% CI 0.77–0.98; Table 3).

**Table 3:** Association between each ocular disease and the risk of major depressive disorder (MDD)

	Number (%)		Risk of MDD	
Ocular Disease	MDD (n = 10,110)	Control (n = 40,440)	OR (95% CI)	*P*
Age-related macular degeneration	195 (1.93)	589 (1.46)	1.33 (1.13–1.57)	**0.001**
Central serous retinopathy	25 (0.25)	78 (0.19)	1.28 (0.82–2.01)	0.280
Retinal vascular occlusion	37 (0.37)	122 (0.30)	1.21 (0.84–1.76)	0.304
Diabetic retinopathy	230 (2.27)	972 (2.40)	0.95 (0.82–1.09)	0.447
Glaucoma	460 (4.55)	1501 (3.71)	1.24 (1.11–1.37)	**< 0.001**
Glaucoma suspect	161 (1.59)	441 (1.09)	1.47 (1.22–1.76)	**< 0.001**
Open-angle glaucoma	138 (1.36)	376 (0.93)	1.47 (1.21–1.80)	**< 0.001**
Closed-angle glaucoma	130 (1.29)	433 (1.07)	1.20 (0.99–1.47)	0.067
Undetermined glaucoma	235 (2.3)	777 (1.9)	1.21 (1.05–1.40)	**0.009**
Dry eye syndrome	994 (9.83)	3326 (8.22)	1.22 (1.13–1.31)	**< 0.001**
Optic neuropathy	14 (0.14)	42 (0.10)	1.33 (0.73–2.44)	0.351
Negative control exposure				
Retinal detachment	35 (0.35)	129 (0.32)	1.09 (0.75–1.58)	0.668
Uveitis	100 (0.99)	338 (0.84)	1.19 (0.95–1.48)	0.138
Blepharitis	336 (3.32)	1545 (3.82)	0.87 (0.77–0.98)	**0.019**

*OR* odds ratio; *CI* confidence interval; *NA* not applicable

### Schizophrenia cohort

The results demonstrated that a presence of previous glaucoma was significantly associated with a higher risk of schizophrenia (OR 1.47, 95% CI 1.02–2.11). Among the different types of glaucoma, glaucoma suspect (OR 1.88, 95% CI 1.01–3.49) and open-angle glaucoma (OR 2.19, 95% CI 1.13–4.26) showed significant associations with schizophrenia. However, no significant associations were observed between other ocular neurovascular diseases or negative control and schizophrenia (Table 4).

**Table 4:** Association between each ocular disease and the risk of schizophrenia

	Number (%)		Risk of schizophrenia	
Ocular Disease	Schizophrenia (n = 1623)	Control (n = 6492)	OR (95% CI)	*P*
Age-related macular degeneration	10 (0.62)	36 (0.55)	1.11 (0.55–2.25)	0.768
Central serous retinopathy	0 (0.0)	8 (0.12)	NA	NA
Retinal vascular occlusion	0 (0.0)	13 (0.20)	NA	NA
Diabetic retinopathy	14 (0.86)	71 (1.09)	0.79 (0.44–1.40)	0.418
Glaucoma	39 (2.40)	107 (1.65)	1.47 (1.02–2.11)	**0.037**
Glaucoma suspect	15 (0.92)	32 (0.49)	1.88 (1.01–3.49)	**0.045**
Open-angle glaucoma	12 (0.74)	22 (0.34)	2.19 (1.13–4.26)	**0.021**
Closed-angle glaucoma	6 (0.37)	29 (0.45)	0.83 (0.34–2.00)	0.673
Undetermined glaucoma	19 (1.2)	46 (0.7)	1.66 (0.98–2.81)	0.059
Dry eye syndrome	78 (4.81)	295 (4.54)	1.06 (0.82–1.37)	0.655
Optic neuropathy	0 (0.0)	3 (0.05)	NA	NA
Negative control exposure				
Retinal detachment	2 (0.12)	13 (0.20)	0.61 (0.14–2.73)	0.523
Uveitis	5 (0.31)	23 (0.35)	0.87 (0.33–2.29)	0.777
Blepharitis	26 (1.60)	137 (2.11)	0.76 (0.50–1.15)	0.188

*OR* odds ratio; *CI* confidence interval, *NA* not applicable

## Discussion

In our study, we found that ocular neurovascular diseases were associated with psychiatric disorders. Glaucoma had the strongest association in all BD, MDD, and schizophrenia cohorts. Additionally, age-related macular degeneration and dry eye syndrome were associated with MDD.

Glaucoma was once considered to be a disease related to elevated intraocular pressure but is now viewed as a neurodegenerative disease [18]. It was also associated with ocular perfusion pressure and vascular dysfunction [19]. A previous study reported that glaucoma was associated with anxiety, depression, and sleep disturbance, and the severity of glaucoma was a predictor of psychiatric disorder [20]. Another large-scale population-based study showed a significant association between glaucoma and anxiety/depression [21]. However, most of those studies consisted of a cross-sectional design and failed to clarify temporality. A previous retrospective study found that veterans with severe mental illness (schizophrenia, schizoaffective disorder, BD, and other psychosis) had an elevated risk of ocular diseases, including cataracts and glaucoma [22]. However, the study did not discuss the pattern of different ocular diseases in each mental illness.

In the present study, we found associations between glaucoma and BD, MDD, and schizophrenia. Among the different types of glaucoma, glaucoma suspect and open-angle glaucoma were associated with all the three target psychiatric disorders. Closed-angle glaucoma was associated with BD and positively correlated with MDD but without statistical significance. Undetermined glaucoma was associated with BD and MDD and also positively correlated with schizophrenia, although this was not statistically significant. Other than glaucoma, age-related macular degeneration and dry eye syndrome were associated with MDD, but this trend was not observed in the BD and schizophrenia groups.

Loss of vision, social, and daily life function has been reported to be associated with increased risk of MDD in glaucoma [23, 24]. In our study, we analyzed additional ocular disorders with potential loss of vision and social function in addition to glaucoma. For example, retinal vascular occlusion is associated with severe vision loss and visual field defect [25]; diabetic retinopathy is among the leading causes of legal blindness in working-age adults and the leading cause of vision-threatening retinopathy [26, 27]. In non-neurovascular exposures, the visual outcome of retinal detachment is uncertain, and surgical interventions for retinal detachment (with attendant postoperative complications) are usually required [28, 29]; uveitis, which causes several vision-threatening complications such as macular edema and cataract, was reported to account for up to 10% of legal blindness in the United States [30, 31]. For these ocular disorders, however, we did not find a significant association with MDD. Thus, psychosocial factors or vision loss may not explain the association between glaucoma and psychiatric disorders identified in our study.

Neurodegenerative process may support the association between glaucoma and psychiatric disorders. One previous study has found that BD and MDD were associated with neurological deficits [3]. Furthermore, glaucoma is characterized by progressive optic nerve degeneration [32] and was recently considered to be a neurodegenerative disease [33]. It has been suggested that the link between mental disorders and glaucoma is attributed to their similar disease pathophysiology. Although the pathogenesis of glaucoma is related to retinal ganglion cell death, caused by intraocular pressure, different types of glaucoma have a slightly different pathophysiology [34]. For example, closed-angle glaucoma is related to ocular structural abnormalities, which leads to elevated intraocular pressure, whereas open-angle glaucoma is more likely to occur in primary neural pathological processes [34]. This may indicate a stronger association between open-angle glaucoma and metal disorders found in our study. As for glaucoma suspect, it is dependent on a normal open angle upon ocular examination and is sometimes defined as an early open-angle glaucoma. In addition to the neurodegenerative hypothesis, glaucoma treatment and its chronic asymptomatic, but potentially blinding nature, may also increase the risk of MDD [21, 35–37].

Regarding dry eye syndrome and age-related macular degeneration, which were both shown to be associated with MDD, there is supporting evidence to our findings. The connection between dry eye syndrome and affective disorders, especially anxiety and MDD, has been discussed [38]. Although the cause and effect relationship remains unknown, similar etiopathogenic and neuropathogenic mechanisms were suggested [38]. As for age-related macular degeneration, it is a potentially distressing medical condition because of the vision loss, financial burden of treatment, and long-term need of intraocular injection [39]. Although the association between age-related macular degeneration and MDD at the neurological level has not been confirmed, one previous study has indicated a possible physiological connection between the diseases [40].

We chose three common ocular diseases, other than neurovascular disease, as our negative controls for exposure, which included retinal detachment, uveitis, and blepharitis. None of the three diseases showed any significant association with any psychiatric disorders, except blepharitis in MDD. Unlike dry eye syndrome or other ocular neurovascular diseases, blepharitis showed a negative association with MDD. As with MDD, blepharitis has been confirmed to be a risk factor for dry eye syndrome and could accelerate the development of the syndrome [38]. In addition, blepharitis has been suggested to be an early form and manifestation of dry eye syndrome [41]. We hypothesize that patients with MDD and blepharitis, both being risk factors for dry eye syndrome, experience early development of dry eye syndrome. Patients may be diagnosed as having and be treated more predominantly for dry eye syndrome than blepharitis in ophthalmology clinics. This may result in a relatively lower prevalence of blepharitis among MDD group compared with its control group.

To the best of our knowledge, this is the first population-based study with long-term follow-ups to test the association between ocular neurovascular diseases and psychiatric disorders. We not only demonstrated the association but also revealed the temporality between the two groups of diseases. We used a large nationwide, population-based sample as our study population and tried to minimize recall bias and selection bias.

There are still some limitations to this study. First, we identified our study groups based on the ICD codes. We attempted to validate the diagnosis by using three similar diagnoses within our study period, which were made by experts (ophthalmologists or psychiatrists). Without a chart review, we were missing each patient’s raw data or other clinical manifestation. Second, psychiatric disorders result from the interactions of bio-psycho-social factors. Our study focused on the biological aspect but cannot evaluate psycho-social confounders between ocular diseases and psychiatric disorders. Third, because the study period included the transition from the ICD-9 and ICD-10 coding system in the Taiwan NHI, we included both ICD-9 and ICD-10 diagnoses, despite the two systems were not perfectly matched for each diagnosis. Fourth, we suggest that patients do not use psychotropic medication before the diagnosis of a psychiatric disorder to rule out the effect of psychotropic medication on the eye; however, in very rare circumstances, they may still use psychotropic medication for other purposes. Finally, we cannot rule out patients who were diagnosed with a psychiatric disorder before our study period but then returned to the NHI system after a long time period without treatment or follow-up. An additional limitation of our study is that patients with prodromal schizophrenia or with untreated psychosis were not be enrolled because the study was based on data collected from a health insurance database. Moreover, the onset of psychiatric disorders, especially schizophrenia, is typically during adolescence or early adulthood. The mean age of our cohort was in the 40s, which means the study enrolled mostly late onset schizophrenia and excluded early onset cases, and thus, the study population cannot represent all the cases of psychiatric disorders. We examined the demographic data of both excluded and included samples of each major psychiatric disorder group and identified no significant difference between the two (Supplementary Table 2). The most common comorbid ocular neurovascular disease among the excluded samples was glaucoma in all groups (BD, MDD, and schizophrenia) (3.02, 4.08, and 1.96%, respectively; Supplementary Table 3).

## Conclusions

Under the hypothesis of the shared pathophysiology of neurovascular dysfunction, our study established the temporality and association between ocular neurovascular diseases and certain psychiatric diseases. Glaucoma, among other ocular neurovascular diseases, had the most significant association of an increased risk for BD, MDD, and schizophrenia. Among the different types of glaucoma, open-angle glaucoma was associated with all three psychiatric disorders, but closed-angle glaucoma was only associated with an increased risk of BD. Additionally, age-related macular degeneration and dry eye syndrome was associated with an increased risk of MDD. Based on the results, we suggest raising awareness of psychiatric disorder during ophthalmology follow-up for glaucoma and of ocular problems during psychiatry follow-up. Appropriate early screening or consultation with another specialty may be indicated. The actual pathophysiology between glaucoma and psychiatric disorder need further investigation. Knowing more about the pathophysiology, may contribute to more knowledge about the mechanism of psychiatric disorder.

## Supplementary information

**Additional file 1: Supplementary Table 1**. ICD-9 CM diagnostic codes used in this study.

**Additional file 2: Supplementary Table 2.** Characteristics of cases according to exclude or not.**Supplementary Table 3.** Ocular disease according to bipolar disorder (BD), major depressive disorder (MDD) and schizophrenia.

## Data Availability

The datasets generated and/or analysed during the current study are not publicly available due legal restriction but are available from the corresponding author on reasonable request.

## Abbreviations

BD: Bipolar disorder
CCI: Charlson Comorbidity Index
GEE: Generalized estimating eq.
ICD-9-CM: International Classification of Diseases, Ninth Revision, Clinical Modification
LHID 2010: Longitudinal Health Insurance Database 2010
MDD: Major depressive disorder
NHIRD: National Health Insurance Research Database
NHI: National Health Insurance
PSM: Propensity score matching
STD: Standardized difference
