# Supplementary material for: Association of ocular diseases with schizophrenia, bipolar disorder, and major depressive disorder: a retrospective case-control, population-based study
Source: BMC Psychiatry. 2020 Oct 2;20:486. doi: 10.1186/s12888-020-02881-w (PMC7532110; doi:10.1186/s12888-020-02881-w)
Supplement: Supplementary file 1 — Additional file 1: Supplementary Table 1. ICD-9 CM diagnostic codes used in this study. [file 12888_2020_2881_MOESM1_ESM.docx]

**Supplementary Table 1**. ICD-9 CM diagnostic codes used in this study

| Variable | ICD-9 CM Code |
| --- | --- |
| Bipolar disorder | 296.0x, 296.1x, 296.4x, 296.5x, 296.6x, 29.68x |
| Substance use | 292.x, 304.x, 305.2-305.9 |
| Alcoholism | 291.x, 303.x, 305.0, 357.5, 425.5, 535.3, 571.0, 571.1, 571.2, 571.3 |
| Major depressive disorder | 296.2x, 296.3x, 311.xx |
| Schizophrenia | 295.xx |
| Anxiety disorder | 300.x (except 300.4) |
| Hypertension | 401.xx-405.xx |
| Dyslipidemia | 272.xx |
| Diabetes | 250.xx |
| Coronary heart disease | 410.xx-414.xx |
| Chronic obstructive pulmonary disease | 491.xx, 492.xx, 496.xx |
| Chronic kidney disease | 580.xx-589.xx, 403.xx-404.xx, 016.0x, 095.4x, 236.9x, 250.4x, 274.1x, 442.1x, 447.3x, 440.1x, 572.4x, 642.1x, 646.2x, 753.1x, 283.11, 403.01, 404.02, 446.21 |
| Stroke | 430.xx-437.xx |
| Age-related macular degeneration | 362.50, 362.51, 362.52 |
| Central serous retinopathy | 362.41 |
| Retinal vascular occlusion | 362.3x |
| Diabetic retinopathy | 362.0x, 362.1x, 362.2x |
| Glaucoma | 365.xx |
| Glaucoma suspect | 365.0x |
| Open-angle glaucoma | 365.1x |
| Closed-angle glaucoma | 365.2x |
| Undetermined glaucoma | 365.4x-365.6x, 365.8x-365.9x |
| Dry eye syndrome | 370.33, 370.43, 372.53, 375.15 and 710.2 |
| Optic neuropathy | 377.1x, 377.3x, 377.4x |
| Retinal detachment | 361.0x |
| Uveitis | 363.0x, 363.1x, 363.2x, 361.0x, 364.1x, 364.2x, 364.3, 379.0x |
| Blepharitis | 373.0x |

ICD-9 CM, International Classification of Diseases, Ninth Revision, Clinical Modification.
