# Supplementary material for: Association of ocular diseases with schizophrenia, bipolar disorder, and major depressive disorder: a retrospective case-control, population-based study
Source: BMC Psychiatry. 2020 Oct 2;20:486. doi: 10.1186/s12888-020-02881-w (PMC7532110; doi:10.1186/s12888-020-02881-w)
Supplement: Supplementary file 2 — Additional file 2: Supplementary Table 2. Characteristics of cases according to exclude or not.Supplementary Table 3. Ocular disease according to bipolar disorder (BD), major depressive disorder (MDD) and schizophrenia. [file 12888_2020_2881_MOESM2_ESM.docx]

**Supplementary Table 2.** Characteristics of cases according to exclude or not

|  | Bipolar Disorder | | |  | Major Depressive Disorder | | |  | Schizophrenia | | |
| --- | --- | --- | --- | --- | --- | --- | --- | --- | --- | --- | --- |
| Variable | Excluded cases  (n = 1,389) | Included cases  (n = 2,243) | STD |  | Excluded cases  (n = 4,832) | Included cases  (n = 10,110) | STD |  | Excluded cases  (n = 4,023) | Included cases  (n = 1,623) | STD |
| Male | 607 (43.7) | 838 (37.4) | 0.13 |  | 1,680 (34.8) | 3,561 (35.2) | -0.01 |  | 2,078 (51.7) | 699 (43.1) | 0.17 |
| Age (years) | 43.9 ± 15.1 | 43.5 ± 16.1 | 0.02 |  | 47.9 ± 15.6 | 47.7 ± 16.9 | 0.01 |  | 42.9 ± 12.6 | 41.9 ± 14.3 | 0.07 |
| Urbanization level |  |  |  |  |  |  |  |  |  |  |  |
| Low | 131 (9.4) | 152 (6.8) | 0.10 |  | 365 (7.6) | 781 (7.7) | -0.01 |  | 510 (12.7) | 174 (10.7) | 0.06 |
| Moderate | 365 (26.3) | 584 (26.0) | 0.01 |  | 1,266 (26.2) | 2,787 (27.6) | -0.03 |  | 1,221 (30.4) | 474 (29.2) | 0.03 |
| High | 466 (33.5) | 768 (34.2) | -0.01 |  | 1,681 (34.8) | 3,373 (33.4) | 0.03 |  | 1,252 (31.1) | 494 (30.4) | 0.01 |
| Very High | 427 (30.7) | 739 (32.9) | -0.05 |  | 1,520 (31.5) | 3,169 (31.3) | <0.01 |  | 1,040 (25.9) | 481 (29.6) | -0.08 |
| Monthly income, NTD |  |  |  |  |  |  |  |  |  |  |  |
| 0–17,880 | 656 (47.2) | 886 (39.5) | 0.16 |  | 1,924 (39.8) | 3,419 (33.8) | 0.12 |  | 2,658 (66.1) | 920 (56.7) | 0.19 |
| 17,881–22,800 | 426 (30.7) | 757 (33.7) | -0.07 |  | 1,536 (31.8) | 3,373 (33.4) | -0.03 |  | 997 (24.8) | 485 (29.9) | -0.11 |
| > 22,800 | 307 (22.1) | 600 (26.7) | -0.11 |  | 1,372 (28.4) | 3,318 (32.8) | -0.10 |  | 368 (9.1) | 218 (13.4) | -0.14 |
| Comorbidity |  |  |  |  |  |  |  |  |  |  |  |
| Anxiety disorder | 805 (58.0) | 1,399 (62.4) | -0.09 |  | 3,746 (77.5) | 5,307 (52.5) | 0.54 |  | 1,459 (36.3) | 655 (40.4) | -0.08 |
| Hypertension | 308 (22.2) | 521 (23.2) | -0.03 |  | 1,420 (29.4) | 2,918 (28.9) | 0.01 |  | 642 (16.0) | 263 (16.2) | -0.01 |
| Dyslipidemia | 243 (17.5) | 409 (18.2) | -0.02 |  | 1,115 (23.1) | 2,252 (22.3) | 0.02 |  | 463 (11.5) | 179 (11.0) | 0.02 |
| Diabetes | 187 (13.5) | 268 (11.9) | 0.05 |  | 672 (13.9) | 1,351 (13.4) | 0.02 |  | 457 (11.4) | 136 (8.4) | 0.10 |
| Coronary heart disease | 149 (10.7) | 262 (11.7) | -0.03 |  | 818 (16.9) | 1,649 (16.3) | 0.02 |  | 232 (5.8) | 112 (6.9) | -0.05 |
| COPD | 155 (11.2) | 203 (9.1) | 0.07 |  | 622 (12.9) | 1,115 (11.0) | 0.06 |  | 331 (8.2) | 113 (7.0) | 0.05 |
| Chronic kidney disease | 91 (6.6) | 151 (6.7) | -0.01 |  | 431 (8.9) | 815 (8.1) | 0.03 |  | 192 (4.8) | 80 (4.9) | -0.01 |
| Stroke | 43 (3.1) | 71 (3.2) | <0.01 |  | 216 (4.5) | 441 (4.4) | 0.01 |  | 75 (1.9) | 47 (2.9) | -0.07 |
| CCI score | 0.54 ± 1.11 | 0.56 ± 1.13 | -0.02 |  | 0.66 ± 1.17 | 0.68 ± 1.24 | -0.02 |  | 0.31 ± 0.78 | 0.39 ± 0.96 | -0.09 |

BD, bipolar disorder; MDD, major depressive disorder; STD, standardized difference; OPD, outpatient clinic; OPH, ophthalmology; NTD, new Taiwan dollar; COPD, chronic obstructive pulmonary disease; CCI, Charlson Comorbidity Index;

Data are presented as the frequency (percentage) or the mean ± standard deviation.

**Supplementary Table 3.** Ocular disease according to bipolar disorder (BD), major depressive disorder (MDD) and schizophrenia

| Ocular Disease | Bipolar Disorder  (n = 1,389) | Major Depressive Disorder  (n = 4,832) | Schizophrenia  (n = 4,023) |
| --- | --- | --- | --- |
| Age-related macular degeneration | 8 (0.58) | 76 (1.57) | 7 (0.17) |
| Central serous retinopathy | 2 (0.14) | 8 (0.17) | 3 (0.07) |
| Retinal vascular occlusion | 1 (0.07) | 18 (0.37) | 2 (0.05) |
| Diabetic retinopathy | 26 (1.87) | 90 (1.86) | 37 (0.92) |
| Glaucoma | 42 (3.02) | 197 (4.08) | 79 (1.96) |
| Glaucoma suspect | 10 (0.72) | 49 (1.01) | 21 (0.52) |
| Open-angle glaucoma | 18 (1.30) | 59 (1.22) | 19 (0.47) |
| Closed-angle glaucoma | 7 (0.50) | 56 (1.16) | 15 (0.37) |
| Undetermined glaucoma | 19 (1.37) | 101 (2.09) | 33 (0.82) |
| Dry eye syndrome | 92 (6.6) | 481 (10.0) | 131 (3.3) |
| Optic neuropathy | 3 (0.22) | 12 (0.25) | 4 (0.10) |
| Any of above | 136 (9.8) | 696 (14.4) | 218 (5.4) |

Data are presented as the frequency (percentage).
